# Bright and photostable push-pull pyrene dye visualizes lipid order variation between plasma and intracellular membranes

**DOI:** 10.1038/srep18870

**Published:** 2016-01-11

**Authors:** Yosuke Niko, Pascal Didier, Yves Mely, Gen-ichi Konishi, Andrey S. Klymchenko

**Affiliations:** 1 Laboratoire de Biophotonique et Pharmacologie, UMR 7213 CNRS, Université de Strasbourg, Faculté de Pharmacie, 74, Route du Rhin, 67401 ILLKIRCH Cedex, France; 2 Department of Organic and Polymeric Materials, Tokyo Institute of Technology, 2-12-1-H-134 O-okayama, Meguro-ku, Tokyo 152-8552, Japan

## Abstract

Imaging lipid organization in cell membranes requires advanced fluorescent probes. Here, we show that a recently synthesized push-pull pyrene (PA), similarly to popular probe Laurdan, changes the emission maximum as a function of lipid order, but outperforms it by spectroscopic properties. In addition to red-shifted absorption compatible with common 405 nm diode laser, PA shows higher brightness and much higher photostability than Laurdan in apolar membrane environments. Moreover, PA is compatible with two-photon excitation at wavelengths >800 nm, which was successfully used for ratiometric imaging of coexisting liquid ordered and disordered phases in giant unilamellar vesicles. Fluorescence confocal microscopy in Hela cells revealed that PA efficiently stains the plasma membrane and the intracellular membranes at >20-fold lower concentrations, as compared to Laurdan. Finally, ratiometric imaging using PA reveals variation of lipid order within different cellular compartments: plasma membranes are close to liquid ordered phase of model membranes composed of sphingomyelin and cholesterol, while intracellular membranes are much less ordered, matching well membranes composed of unsaturated phospholipids without cholesterol. These differences in the lipid order were confirmed by fluorescence lifetime imaging (FLIM) at the blue edge of PA emission band. PA probe constitutes thus a new powerful tool for biomembrane research.

Cell membranes exhibit inhomogeneous lipid distribution at different levels. The first one is related to the lateral heterogeneity in cell membranes, which is described by the lipid raft hypothesis^1^. It has been a subject of intensive research and debates in the last decade^2^,^3^,^4^,^5^,^6^,^7^,^8^,^9^,^10^,^11^,^12^. In model membranes, the lipid rafts are observed as separate rigid domains (liquid ordered phase, (Lo)) formed of saturated lipids and cholesterol, coexisting with liquid disordered phase (Ld) domains consisting of unsaturated lipids. Lipid rafts are believed to be behind many membrane and/or cellular processes such as formation of protein clusters, signal transduction, pathogen invasion, cholesterol homeostasis, neurodegenerative diseases, and angiogenesis^1^,^2^,^3^,^4^,^5^,^6^,^7^,^8^,^9^,^10^,^11^,^12^. Transmembrane lipid asymmetry is the second important heterogeneity in biomembranes. It is known that in healthy cells, the outer leaflet contains mainly sphingomyelin and phosphatidyl choline, while the inner one is represented by phosphatidyl ethanolamine and phosphatidyl serine^13^. This asymmetry is lost in programmed cell death (apoptosis)^14^. The third, and the least explored heterogeneity is related to the differences in lipid composition and organization between the cell plasma membrane and the intracytoplasmic (intracellular) membranes. It is well known that >60% of all cellular cholesterol is localized at the plasma membrane, so that the intracellular membranes, such as endoplasmic reticulum and nuclear membrane are poor in cholesterol^15^,^16^. Indeed, the concentration of cholesterol in plasma membranes can reach 40%, while in endoplasmic reticulum it can be as low as 5%^16^.

A variety of techniques have been applied so far to explore the lipid organization, including atomic force microscopy^17^,^18^,^19^,^20^,^21^,^22^, nuclear magnetic resonance^23^,^24^, and mass spectrometry^25^. In particular, fluorescence techniques have attracted considerable attention since they can be used to noninvasively study model membranes and living cells, though the performance of these techniques largely rely on the fluorescent probes employed^8^. Among existing probes, the solvatochromic ones, such as Laurdan^26^,^27^,^28^,^29^ and its derivatives^30^,^31^, have been largely used for probing lipid rafts due to their ability to distinguish Lo from Ld domains by changing fluorescence color, intensity, and lifetime. Being a typical polarity-sensitive dye, Laurdan shifts its emission maximum in response to changes in membrane hydration and solvent relaxation^27^,^32^,^33^, which are parameters linked to lipid order. However, Laurdan absorbs in the UV range, so that its application in cellular imaging requires expensive two-photon excitation source. Moreover, similarly to many solvatochromic dyes, the photostability of Laurdan in apolar media, relevant to lipid membranes, is limited^31^. Recently developed analogues of Laurdan with extended conjugation showed promising characteristics^34^,^35^,^36^,^37^,^38^, though their application to study lipid organization in model and cell membranes was not explored.

Recently, we developed new solvatochromic push-pull fluorophores based on pyrene^34^,^35^. Among them, **PA** (Fig. 1) shows the most attractive features such as high brightness (absorption coefficient is 25000 M^−1^ cm^−1^ in ethanol and fluorescence quantum yields, QY = 0.93 and 0.77 in hexane and ethanol, respectively), absorption band in the visible region (~420 nm), and strong solvatochromism (red shift from 480 nm to 598 nm on solvent change from hexane to ethanol)^35^. These photophysical properties of **PA** in organic solvents are superior to those of the commonly used Laurdan and Prodan derivatives, so that **PA** appears as a promising alternative membrane probe for lipid domains. Moreover, its hydrophobic nature is expected to make it cell-permeable, which should enable probing both plasma and intracellular membranes.

In this work, we report the fluorescence behavior of **PA** in several lipid model vesicles in comparison to Laurdan. As expected, **PA** showed clear advantages over Laurdan, that is, red-shifted absorption compatible with common diode laser (405 nm) source and bright fluorescence with large Stokes shift even in model membranes. Importantly, **PA** also exhibits a much higher photostability than Laurdan, even though low photostability is a frequent concern for solvatochromic dyes^8^. On the other hand, similarly to Laurdan, it readily distinguishes between Lo and Ld phases by its emission color, so that **PA** appears as a lipid raft probe. Finally, cellular studies showed that **PA** binds biomembranes at the cell surface and inside the cells. Remarkably, **PA** revealed a clear change in the lipid order of cell membranes which decreased from the plasma membrane towards the nucleus.

## Results and Discussion

**Figure 2:**
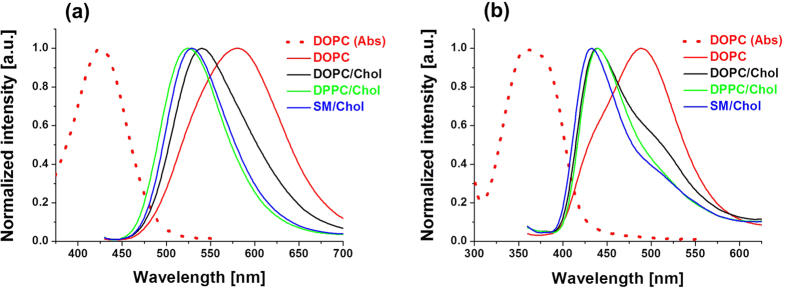
Spectroscopic response of PA and Laurdan to lipid order. Normalized absorption and fluorescence spectra of (**a**) **PA** and (**b**) Laurdan in LUVs of different composition and lipid order. The concentration of probes and lipids were 1 μM and 200 μM, respectively. Excitation wavelengths for PA and Laurdan were 430 nm and 360 nm, respectively.

The absorption and fluorescence spectra of **PA** and Laurdan were recorded in various model lipid vesicles (large unilamellar vesicles, LUVs). As shown in Fig. 2 and Table 1, the absorption maximum of **PA** in LUVs was around 430 nm, which was ~70 nm red-shifted as compared to Laurdan. This allows **PA** excitation with a common 405 nm laser source, in contrast to Laurdan that requires a two-photon (TP) excitation. The Stokes Shifts of **PA** were relatively large being comparable to those of Laurdan (generally smaller in wavenumbers, but larger in the wavelength scale, Table 1), so that the emission band of **PA** was strongly red-shifted compared to Laurdan for all studied lipid compositions. A large Stokes shift with the red shifted emission is helpful to decrease the autofluorescence in biological samples, especially in the blue region of the visible spectrum. Moreover, **PA** exhibited higher fluorescence quantum yield than Laurdan. Taking into account also its higher extinction coefficient (25000 and 20000 M^−1^cm^−1^ for **PA**^35^ and Laurdan (supplier data), respectively, in alcohols), **PA** is significantly brighter than Laurdan. On the other hand, similarly to Laurdan, **PA** exhibited strong shifts in its emission band in response to the changes in the composition and the phase state of LUVs. The emission wavelength of **PA** depended strongly on the lipid order, so that in the Lo phase the emission spectrum was blue shifted with respect to that in Ld phase (Fig. 2, Table 1). Remarkably, **PA** and Laurdan showed comparable spectral shifts in response to changes in the lipid order. Observed tendency of spectral shift in LUVs is in line with the results obtained with other solvatochromic dyes^26^,^27^,^28^,^29^,^30^,^31^,^39^,^40^,^41^,^42^,^43^,^44^,^45^, indicating that **PA** can correctly reflect the less polar and less hydrated environment in the Lo phase and therefore work as a lipid order indicator. However, the emission of **PA** in DOPC vesicles (Ld) was strongly blue-shifted in the presence of cholesterol, decreasing the difference between Ld phase with cholesterol and Lo phase. In fact, Laurdan showed exactly the same behavior, so that our **PA** probe is suitable at least for those membrane applications where Laurdan was successfully used. As the full width at half maximum (FWHM) of **PA** was comparable to that of Laurdan in different LUVs (generally smaller in wavenumbers, but larger in the wavelength scale, Table 1), we could consider that the phase sensitivity of these two dyes should be similar. To make a more precise estimation, we compared their ratiometric response to the lipid order. To this end, we calculated the integral intensity ratio of red to blue parts of the emission band split at 455 and 550 nm for Laurdan and **PA**, respectively (Table 1). It was found that the increase in the Red/Blue ratio from Lo to Ld phase was slightly higher for **PA** as compared to Laurdan.

**Figure 3:**
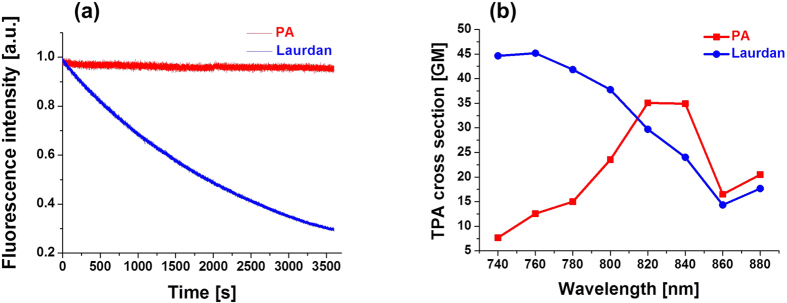
Photostability and two-photon cross sections of **PA** and Laurdan. (**a**) Photodegradation as a function of time for 1 μM **PA** and Laurdan in lipid vesicles composed of 200 μM DOPC in HEPES 20 mM, pH = 7.4. Photodegradation was performed using a 430 nm and 360 nm excitation wavelength for **PA** and Laurdan, respectively, with the xenon lamp in the spectrofluorometer (*ε*_PA_*I*_430_/*ε*_Lau_*I*_360_ = 2:1. *ε*_PA_ and *ε*_Lau_ denote absorption coefficient of **PA** at 430 nm (25000) and Laurdan at 360 nm (18400), respectively; *I*_430_ and *I*_360_ denote the excitation power at 430 nm and 360 nm, respectively). (**b**) Two-photon absorption (TPA) cross sections of **PA** and Laurdan in ethanol obtained by using rhodamine B as a standard^58^,^59^.

One remarkable feature of **PA** was found to be its photostability in lipid vesicles. As shown in Fig. 3a, **PA** in DOPC lipid vesicles maintained about 99% of its fluorescence intensity after continuous photoexcitation for 1 h, whereas for Laurdan the remaining fluorescence intensity was <30%. This high photostability is remarkable, as low photostability is a crucial drawback for most solvatochromic fluorophores. According to Branchard *et al.*^46^, photobleaching is often triggered in the triplet excited state of the fluorophore. As this state has quite a long lifetime compared to the singlet state one, electron and/or energy transfer can occur from the triplet state to the molecular oxygen. This process generates superoxide radical and/or singlet oxygen, which are reactive enough to cause dye degradation. Therefore, the lower triplet state generation (i.e. slower intersystem crossing), the higher photostability. This problem is especially critical in lipid membranes, because according to the El-Sayed’s rule, apolar media efficiently generate *n* − π* first singlet state prone to intersystem crossing^47^. The fluorescence quantum yields of Laurdan in apolar toluene and cyclohexane were 0.47 and 0.03, respectively, whereas for **PA** they were 0.96 and 0.93 in toluene and hexane, respectively^35^, indicating that the intersystem crossing is much less efficient for **PA** than for Laurdan. In fact, Prodan, a homologue of Laurdan showing identical photophysical properties^48^, was proposed to undergo intersystem crossing in apolar solvents^49^, which was recently supported by DFT calculation^35^. Probably, due to the much larger electronic conjugation of pyrene in **PA**, the π − π* electronic transition remains more energetically favorable than the *n* − π* one even in apolar media, which is not the case for the much smaller naphthalene π−system in Laurdan. This may explain the much higher photostability of **PA**, as compared to Laurdan in the apolar medium of lipid membranes. Given that a majority of solvatochromic fluorophores show decreased fluorescence quantum yields in both apolar and polar solvents^36^,^37^,^38^, **PA** can be considered as an exceptional environment-sensitive probe with high photostability and efficient emission in different media.

In order to evaluate the applicability of **PA** to two-photon (TP) microscopy, the TP absorption cross section of **PA** was recorded with rhodamine B as a reference. As shown in Fig. 3b, **PA** exhibited an absorption maximum (λ_TP,abs_) at around 820 nm. The maximum absorption cross section of **PA** (35 GM) was lower than that of Laurdan (45 GM), but **PA** was more efficient in the region >800 nm, which is more common in TP bioimaging.

Taken together, our data indicate that **PA** appears superior to Laurdan as a membrane probe in terms of excitation in the visible range (or NIR TP excitation >800 nm), brightness, and especially photostability.

**Figure 4:**
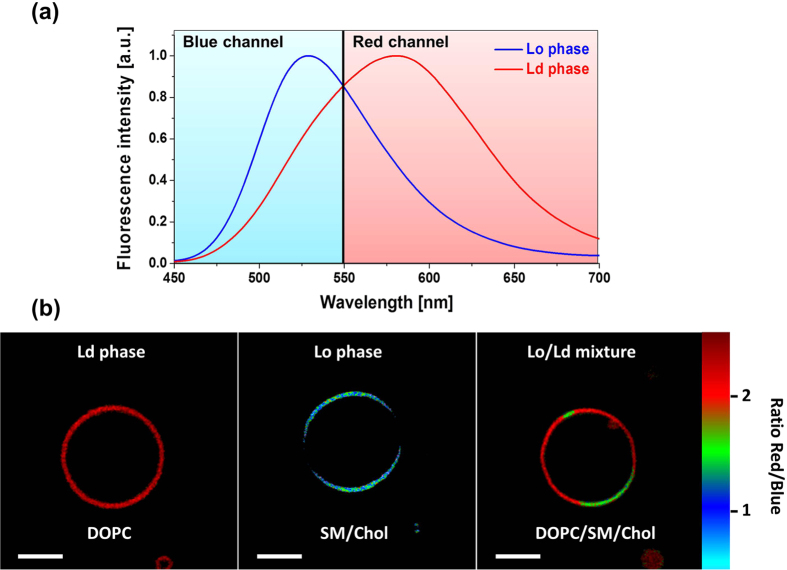
Two-photon excitation microscopy of lipid phases in giant vesicles. (**a**) Normalized fluorescence spectra of **PA** in LUVs of Ld (DOPC) and Lo (SM/Chol) phase. The cyan and magenta regions separated at 550 nm represent the detection range for the blue and red channels of the microscope. (**b**) Fluorescence microscopy imaging of Ld, Lo, and mixed Lo/Ld phases in GUVs stained with **PA**. GUVs were composed of DOPC (Ld), SM/Chol (2/1) (Lo), and DOPC/SM/Chol (1/1/0.5) (Lo/Ld mixture). A dichroic mirror at 550 nm was used to split the emission into the two channels. TP-excitation wavelength was at 830 nm. Probe concentration was 100 nM. Scale bar is 10 μm.

Next, we performed TP excitation fluorescence microscopy imaging of giant unilamellar vesicles (GUVs) stained with **PA** in order to evaluate its capability to visualize Ld and Lo phases. As can be seen in Fig. 4, **PA** distinguished well the Lo and Ld phases through strong changes in the red/blue ratio signal. Similarly, separated domains were clearly observed in GUVs of ternary mixture composition. It should be noted that the Lo phase became more greenish in the ternary mixture as compared to the SM/chol GUVs (Fig. 4b), likely due to some redistribution of cholesterol from the Lo to the Ld phase, which was also observed with other probes, including Laurdan^8^.

**Figure 5:**
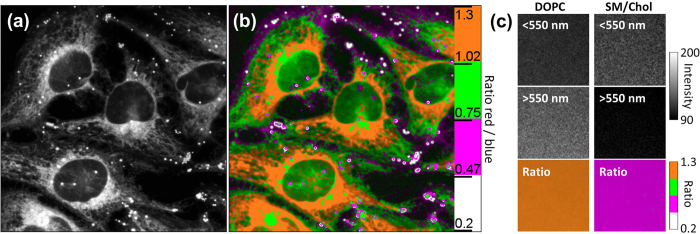
Imaging lipid order in living HeLa cells. Fluorescence intensity (**a**) and ratio (**b**) imaging of HeLa cells stained with 100 nM of **PA**. One-photon laser excitation at 405 nm was employed. The pseudo-colors represent the ratio of the long- to short-wavelength emission channels (550–700 nm to 470–550 nm). The intensity at each pixel represents the sum of intensities at both channels. The scale bar is 20 μm. (**c**) Calibration of the ratio using suspension of lipid vesicles (LUVs) composed of DOPC (Ld phase) and SM/Chol (Lo phase). The top panels present the ratio images.

Finally, we incubated the **PA** dye with HeLa cells in order to evaluate its potential for ratiometric imaging of lipid organization. The emission was detected at two channels, which corresponded to the left and right parts of the emission spectrum of the dye (Fig. 4a), as it was for TP imaging of giant vesicles. Already after 10 min of incubation with 100 nM **PA**, the fluorescence was observed all over the cells (Fig. 5). The fluorescence images indicated that different lipid-containing cellular compartments were stained by the dye. These compartments appeared in the ratiometric image in different pseudo-colors, with lipid droplets being in white, cell plasma membrane in magenta, cytoplasm (presented by endoplasmic reticulum and other membrane-containing organelles) in green – orange and finally nuclear membrane in orange. The fluorescence intensity inside nucleus was low evidently because it does not contain any lipid structures (the observed residual fluorescence in green is likely due to out of focus fluorescence coming from below/above the nucleus). The large variation in pseudo-colors for the different parts of the cells suggests that they are characterized by different local polarity, thereby indicating differences in the lipid organization of these compartments. Thus, the polarity increased in the following order: lipid droplets < plasma membrane < intracellular membranes ≤ nuclear membranes. The low polarity of lipid droplets can be attributed to their high content in triglycerides and cholesteryl esters, which are the least polar lipids in the cell^15^,^16^. More surprising was the difference in polarity between the plasma membranes and the intracellular membranes. To interpret it in terms of lipid composition and lipid order, we recorded ratiometric images of suspensions of DOPC and SM/Chol lipid vesicles (LUVs, ~100 nm diameter) using identical instrumental settings and the same color scale (Fig. 5c). In DOPC vesicles (Ld phase) **PA** displayed nearly doubled red/blue ratio compared to SM/Chol vesicles (Lo phase), in line with the strong band shifts observed in spectroscopy (Fig. 4a) as well as ratio changes in GUVs (Fig. 4b). Remarkably, the magenta pseudo-color obtained for SM/Chol vesicles matched perfectly that in plasma membranes, while orange pseudo-color in DOPC vesicles corresponded to the intracellular membranes close to the nucleus (Fig. 5b,c). Thus, **PA** probe reveals that the cell plasma membranes are mainly presented by Lo phase, while the intracellular membranes are much less ordered being close to Ld phase of DOPC vesicles. Plasma membrane is known to contain significant amounts of sphingomyelin and cholesterol, responsible for the formation of Lo phase^2^,^3^,^4^,^5^,^6^,^7^,^8^,^9^,^10^,^11^,^12^. Moreover, recent works based on Laurdan, di-4-ANEPPDHQ^29^, NR12S^45^,^50^ and F2N12S^51^ probes suggested that Lo phase may dominate the cell plasma membrane. On the other hand, intracellular membranes, especially endoplasmic reticulum, contain much less cholesterol and nearly no sphingomyelin^16^, which could explain their much lower lipid order. This contrast between the plasma membrane and the cytoplasm is in line with a recent report based on Laurdan^28^,^52^, showing that plasma membranes are characterized by higher generalized polarization values. The latter was attributed, according to lifetime measurements, to higher cholesterol content in plasma membrane as compared to intracellular membranes^52^. However, we should stress that our images revealed more details, evidencing the low polarity of lipid droplets as well as the gradual increase in membrane polarity (decrease in the lipid order) from plasma membrane towards the nucleus. To our knowledge, this polarity gradient has never been described before. Its presence could be explained by the gradient of sterol concentration from the endoplasmic reticulum towards plasma membrane shown previously^16^,^53^,^54^. However, we cannot exclude that the signal from the cytoplasm at the cell extremities (appeared in green, Fig. 5b), suggesting lower polarity, was contaminated by the out of focus fluorescence from the plasma membrane located above and below the regions of interest, so that a more detailed study is needed. Importantly, **PA** was used at >20-fold lower concentration (100 nM) than in the previous reports on Laurdan (1.8 μM^52^ or 5 μM^28^) and required a common and inexpensive single-photon light source at 405 nm. The obtained results further highlight the advantages of the new probe as compared to Laurdan.

To confirm the observed differences in the lipid order between plasma membrane and intracellular membranes, we realized fluorescence lifetime imaging (FLIM) experiments on **PA** in HeLa cells. Using two-photon FLIM imaging setup, we first measured average fluorescence lifetime in suspensions of LUVs presenting Ld (DOPC) and Lo (SM/Chol) phases. In Lo phase, **PA** displayed fluorescence lifetime of 6.7 ns, while in Ld phase, it was 4.6 ns. However, we found this difference insufficient, so that the FLIM cellular imaging was not conclusive (data not shown). To improve the contrast in FLIM imaging, we introduced 510-nm band pass filter (FWHM = 20 nm) at the detection channel. Solvatochromic dyes in lipid membranes undergo solvent relaxation process at the nanosecond time scale, so that their emission maximum shifts to the red during the excited-state lifetime^32^,^33^. As the relaxation process depends strongly on the phase state^55^, we expected that the blue part of the emission band may show larger variations in the lifetime than the whole band. Indeed, according to our measurements in LUVs with the emission filter, the average lifetimes of **PA** in Lo and Ld phases were 6.8 and 2.2 ns, respectively. Thus, the emission filter strongly decreased fluorescence lifetime in Ld phase without changing Lo phase, which is expected because in Ld phase efficient solvent relaxation should contribute to faster emission decay at the blue part of **PA** fluorescence band.

**Figure 6:**
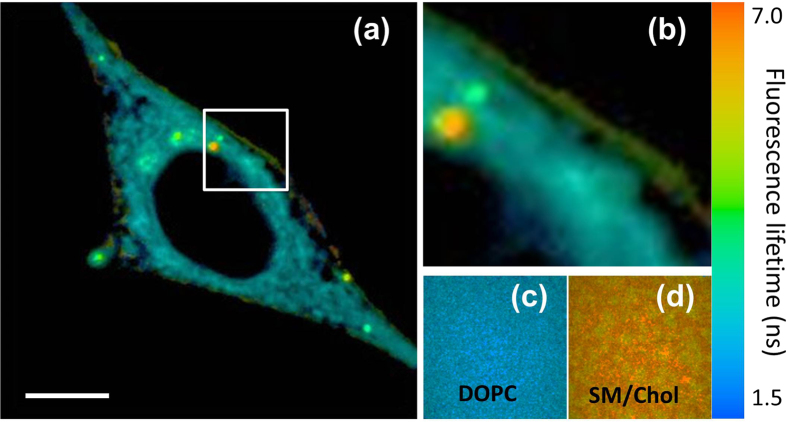
FLIM imaging of HeLa cells stained with PA probe. (**a**) FLIM image of Hela cells and (**b**) zoom at the region where both plasma and intracellular membranes are well seen. Calibration images of suspensions of DOPC (**c**) and SM/Chol (**d**) vesicles (LUVs) recorded with the same instrumental settings. Two-photon excitation wavelength was 830 nm. A band pass filter at 510 nm (FWHM = 20 nm) was used at the detection channel. Scale bar is 10 μm.

In FLIM images of HeLa cells (with the emission filter) stained with **PA**, the cytoplasm appeared in blue (with lifetime ~ 3 ns), which matched perfectly the image obtained for DOPC LUVs (Fig. 6). This means that the intracellular membranes are close to Ld phase, as suggested by our ratiometric measurements (Fig. 5). By contrast, cell plasma membrane appeared in yellow-green pseudo-color, indicating much longer lifetime (~4.5 ns) that matched well to the FLIM data obtained in LUVs of Lo phase. Thus, in line with our ratiometric data (Fig. 5), cell plasma membrane is much more ordered than intracellular membranes. Moreover, these data confirmed that the cell plasma membrane is close by its properties to model vesicles presenting Lo phase.

## Conclusion

In this work, we have demonstrated an application of solvatochromic push-pull pyrene fluorophore, **PA**, to imaging of lipid membranes. Similarly to the commonly used Laurdan, **PA** shows strong variation of its emission color as a function of the lipid order. Importantly, **PA** exhibits a number of key advantages over Laurdan: absorption band in visible region, higher brightness, and red-shifted emission. Furthermore, two-photon absorption cross section of **PA** is sufficiently high for two-photon excitation fluorescence microscopy, which reveals that **PA** can distinguish separated domains of ordered and disordered phases in GUVs. The remarkable result obtained here is much higher photostability of **PA** compared to Laurdan in apolar lipid environment, which might be due to inefficient intersystem crossing. Therefore, we expect **PA** to be less phototoxic (due to lower production of singlet oxygen by the triplet state) than Laurdan or other environment-sensitive dyes in apolar lipid environment. **PA** showed excellent staining of plasma and intracellular membranes of Hela cells. The required concentration of **PA** was >20-fold lower than that commonly used for Laurdan, probably due its higher brightness. Importantly, ratiometric fluorescence imaging with **PA** could reveal a strong variation of the lipid order from the plasma membrane to the cell interior, which, according to Laurdan-based studies, is connected with the uneven distribution of cholesterol. Our data showed that cell plasma membranes are close to the liquid ordered phase of model membranes composed of sphingomyelin and cholesterol, while intracellular membranes are much less ordered, matching well membranes composed of unsaturated phospholipids without cholesterol. These differences in the lipid order between plasma and intracellular membranes were confirmed by FLIM at the blue edge of **PA** emission band. To the best of our knowledge, such detailed and clear observation of lipid order heterogeneity using ratiometric imaging in the cell has never been reported. This high quality imaging is achieved largely due to the excellent photophysical properties of **PA** and its high sensitivity to lipid order. This probe opens new opportunities in ratiometric imaging of lipid organization in the plasma and inner membranes of cells in culture and tissues with high level information content.

## Methods

### Materials

Dioleoylphosphatidylcholine (DOPC), dipalmitoylphosphatidylcholine (DPPC) and cholesterol were purchased from Sigma-Aldrich. Bovine brain sphingomyelin (SM) was from Avanti Polar Lipids (Alabaster, USA). Laurdan was purchased from Life Technologies. Dye **PA** was synthesized as described elsewhere^35^.

### Instrumental

Absorption spectra were recorded on a Cary 4000 spectrophotometer (Varian). Fluorescence spectra were recorded on a FluoroLog (Jobin Yvon, Horiba) spectrofluorometer. The instrument provides correction for the lamp intensity fluctuation and the photo-multiplier sensitivity. Fluorescence emission spectra were systematically recorded at 20 °C, unless indicated. All the spectra were corrected from the fluorescence of the corresponding blank (suspension of lipid vesicles without the probe) and for the wavelength-dependent response function of the detector. Relative fluorescence quantum yields for **PA** and Laurdan in vesicles, toluene and cyclohexane were measured using ethanol solutions of **PA** (QY = 0.77) and Prodan (QY = 0.95) as standard, respectively^35^. In the photostability assays, a 1 μM of dye in 200 μM DOPC (HEPES 20 mM, pH 7.4) in a quartz micro-cuvette (50 μL volume) was illuminated by the Xenon lamp of a FluoroLog spectrofluorometer (excitation slits were opened to 8 nm). The excitation wavelength was a 430 and 360 nm for **PA** and Laurdan, respectively. The illumination power density was ~1.0 and 0.7 mW cm^−2^ for **PA** and Laurdan, respectively. During the time of illumination, the fluorescence intensity maximum was recorded as a function of time.

Two-photon fluorescence microscopy imaging of GUVs was performed by using a home-built two-photon laser scanning setup based on an Olympus IX70 inverted microscope with an Olympus 60 × 1.2NA water immersion objective^56^,^57^. Two-photon excitation was provided by an InSight DS + (680–1300 nm, 120 fs, 80 MHz, Spectra Physics) with an excitation power around 5 mW (830 nm), and photons were detected with Avalanche Photodiodes (APD SPCM-AQR-14-FC, Perkin-Elmer) connected to a counter/timer PCI board (PCI6602, National Instrument). Imaging was carried out using two fast galvo mirrors in the descanned fluorescence collection mode. Images corresponding to the blue and red channels were recorded simultaneously using a dichroic mirror (Beamsplitter 550 nm DCXR) and two APDs. For FLIM measurements, photons were detected through a band pass emission filter centered at 510 nm (FWHM = 20 nm) using an Avalanche Photodiode (APD SPCM-AQR-14-FC, Perkin-Elmer) connected to a TCSPC system (SPC830 Becker-Hickl). Typical acquisition time was 30 s with an excitation power around 5 mW (830 nm) at the laser output. Subsequent data analysis using a commercial software program (SPC Image) allowed us extracting the fluorescence lifetimes from the decays, and their visualization in a FLIM image, using an arbitrary color scale. Confocal microscopy images of cells were taken on a Leica TSC SPE confocal microscope. The excitation light was provided by a 405 nm laser while the fluorescence was detected at two spectral ranges: 470–550 (blue channel) and 550–700 (red channel). The ratiometric images were generated by using special macros under Image J that divides the image of the red channel by that of the blue channel. For each pixel, a pseudo-color scale is used for coding the ratio, while the intensity is defined by the integral intensity recorded for both channels at the corresponding pixel^39^,^40^,^41^.

Two-photon absorption cross section measurements were performed using Rhodamine B in methanol as a calibration standard according to the method of Webb *et al.*^58^,^59^. Two-photon excitation was provided by an InSight DS + (Spectra Physics) with a pulse duration of 120 fs. The laser was focused by an achromatic lens (*f* = 2 cm) in a cuvette containing the dye (70–100 mM in ethanol) and the spectra were recorded with a fibered spectrometer (Avantes) by collecting the fluorescence emission at 90° with a 20 × Olympus objective.

### Lipid vesicles

LUVs were obtained by the extrusion method as previously described^60^. Briefly, a suspension of multilamellar vesicles was extruded by using a Lipex Biomembranes extruder (Vancouver, Canada). The pore size of the filters was first 0.2 μm (10 passages) and thereafter 0.1 μm (10 passages). This generates monodisperse LUVs with a mean diameter of 0.11 μm as measured with a Malvern Zetamaster 300 (Malvern, U.K.). For the absorption and fluorescence spectra performed with LUVs labeled by **PA** or Laurdan, the concentration ratio of the dye to LUVs was 1:200. GUVs were generated by electroformation in a home-built liquid cell (University of Odense, Denmark), using previously described procedures^39^. A 0.1 mM solution of lipids in chloroform was deposited on the platinum wires of the chamber, and the solvent was evaporated under vacuum for 30 min. The chamber thermostated at 55 °C was filled with a 300 mM sucrose solution, and a 2-V, 10-Hz alternating electric current was applied to this capacitor-like configuration for ca. 2 h. Then, a 50 μL aliquot of the obtained stock solution of GUVs in sucrose (cooled down to room temperature) was added to 200 μL of 300 mM glucose solution to give the final suspension of GUVs used in microscopy experiments. The staining of GUVs was performed by addition of the probe in DMSO stock solution to obtain a 100 nM final probe concentration (final DMSO volume is less than 0.25%).

HeLa cells (ATCC) were cultured in Dulbecco’s modified Eagle medium (D-MEM, Low glucose, +GlutaMAX, Gibco-Invitrogen) supplemented with 10% (v/v) fetal bovine serum (FBS, Lonza), and 1% antibiotic solution (penicillin–streptomycin, Gibco-invitrogen) in a humidified incubator with 5% CO_2_ atmosphere at 37 °C. Cells cultures passages were realized every 2–3 days. For microscopy studies with the probes, attached HeLa cells were washed two times by gentle rinsing with HBSS. Then, a freshly prepared solution of **PA** in HBSS was added to the cells to a final probe concentration of 100 nM (<0.25% DMSO volume) and incubated for 7 min in the dark at RT. The obtained samples were imaged directly without washing.

## Additional Information

**How to cite this article**: Niko, Y. *et al.* Bright and photostable push-pull pyrene dye visualizes lipid order variation between plasma and intracellular membranes. *Sci. Rep.* **6**, 18870; doi: 10.1038/srep18870 (2016).

## Figures and Tables

**Figure 1:**
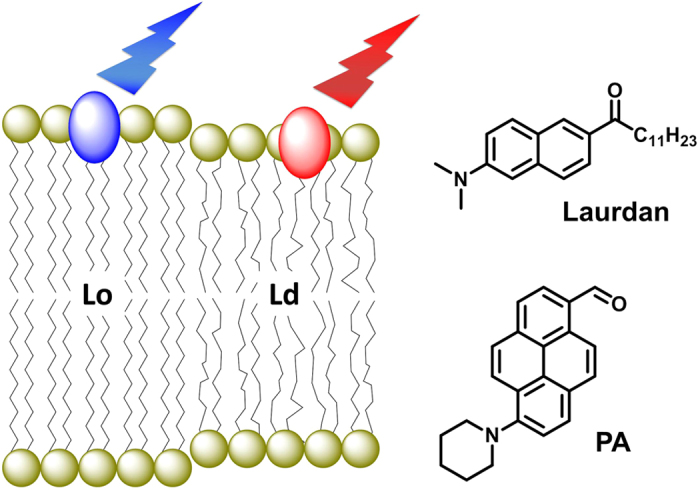
Schematic presentation of color response of solvatochromic fluorescent probes to lipid order in membranes and chemical structures of Laurdan and PA.

**Table 1:** Spectroscopic properties of PA and Laurdan in different lipid vesicles.

Composition	Phasestate	Dye											
		PA						Laurdan					
		λ_abs_nm	λ_em_nm	SS cm^−1^(nm)	FWHM cm^−1^(nm)	R/B	QY	λ_abs_nm	λ_em_nm	SS cm^−1^(nm)	FWHM cm^−1^(nm)	R/B	QY
DOPC	Ld	425	581	6523 (156)	3603 (122)	2.77	62	356	488	7847 (132)	4394 (109)	3.38	25
DOPC/Chol	Ld	428	540	5049 (112)	3516 (100)	1.23	56	358	439	5309 (81)	5451 (98)	1.58	21
DPPC	L*β*	428	537	5016 (109)	3647 (103)	1.16	42	359	444	5484 (85)	3495 (65)	1.27	20
DPPC/Chol	Lo	426	524	4606 (98)	3102 (83)	0.57	60	358	440	5360 (82)	4354 (73)	1.37	17
SM	L*β*	430	545	5140 (95)	3555 (104)	1.27	59	356	433	5101 (77)	4587 (89)	1.16	23
SM/Chol	Lo	431	528	4440 (97)	3004 (82)	0.71	63	357	435	5489 (78)	5366 (97)	1.30	19

^a^λ_abs_ and λ_em_ are the wavelength of absorption and emission maxima, respectively; SS and FWHM denote Stokes shift and full width at half maximum of emission spectra, respectively; R/B is the integral intensity ratio of red to blue parts of the emission band spit at 455 and 550 nm for Laurdan and **PA**, respectively; QY is the fluorescence quantum yield.
